# Austrian consensus guidelines on the management and treatment of portal hypertension (Billroth III)

**DOI:** 10.1007/s00508-017-1262-3

**Published:** 2017-10-23

**Authors:** Thomas Reiberger, Andreas Püspök, Maria Schoder, Franziska Baumann-Durchschein, Theresa Bucsics, Christian Datz, Werner Dolak, Arnulf Ferlitsch, Armin Finkenstedt, Ivo Graziadei, Stephanie Hametner, Franz Karnel, Elisabeth Krones, Andreas Maieron, Mattias Mandorfer, Markus Peck-Radosavljevic, Florian Rainer, Philipp Schwabl, Vanessa Stadlbauer, Rudolf Stauber, Herbert Tilg, Michael Trauner, Heinz Zoller, Rainer Schöfl, Peter Fickert

**Affiliations:** Division of Gastroenterology & Hepatology, Department of Internal Medicine III, Medical University of Vienna, Währinger Gürtel 18–20, 1090 Vienna, Austria

**Keywords:** Portal hypertension, Billroth, Austria, Guidelines, Cirrhosis, Varices, Ascites, TIPS

## Abstract

The Billroth III guidelines were developed during a consensus meeting of the Austrian Society of Gastroenterology and Hepatology (ÖGGH) and the Austrian Society of Interventional Radiology (ÖGIR) held on 18 February 2017 in Vienna. Based on international guidelines and considering recent landmark studies, the Billroth III recommendations aim to help physicians in guiding diagnostic and therapeutic strategies in patients with portal hypertension.

## Chapters of Billroth III

I.: Definitions of portal hypertension
II.: Diagnosis and screening of portal hypertension
III.: Preprimary prophylaxis and prevention of decompensation
IV.: Primary prophylaxis of variceal bleeding
V.: Acute variceal bleeding
VI.: Secondary prophylaxis of variceal bleeding
VII.: Measurement of the hepatic venous pressure gradient (HVPG)
VIII.: Portal hypertensive gastropathy
IX.: Gastric varices
X.: Management of ascites
XI.: Spontaneous bacterial peritonitis (SBP)
XII.: Management of hepatorenal syndrome (HRS-AKI)
XIII.: Transjugular intrahepatic portosystemic shunt (TIPS)
XIV.: Portal vein thrombosis (PVT)

**Table 1:** Grading of evicence (*)

*Evidence*	*Definition*
A—high	Further research is very unlikely to change our confidence in the estimate of effect
B—moderate	Further research is likely to have an important impact on our confidence in the estimate of effect and may change the estimate
C—low	Further research is very likely to have an important impact on our confidence in the estimate of effect. Any estimate of effect is uncertain
*Recommendation*	*Notes*
1—strong	Factors influencing the strength of the recommendation include the quality of evidence, presumed patient-important outcomes, and costs
2—weak	Variability in preferences and values, or more uncertainty, higher cost or resource consumption: a weak recommendation is warranted

*The strength of evidence (high A, moderate B, weak C) and of recommendation (strong 1, weak 2) was based on a modified GRADE system as suggested by the international GRADE group [1]

## I. Definitions of portal hypertension

1. The term compensated advanced chronic liver disease (cACLD) may be used similar to cirrhosis and is defined as confirmed liver stiffness >15 kPa on transient elastography [2]. Diagnosis of cACLD should trigger screening for clinically significant portal hypertension (CSPH) [3]. (A1)
2. CSPH is defined as an increase of the hepatovenous pressure gradient (HVPG) to values of ≥10 mm Hg. (A1) [3]
3. Normal portal pressure is defined as HVPG of ≤5 mm Hg, while subclinical portal hypertension is defined as HVPG 6–9 mm Hg. (A1)
4. CSPH might already be present in compensated patients (without ascites, without varices). (A1)
5. The presence of gastroesophageal varices (GOVs), variceal hemorrhage, ascites (in the absence of significant cardiac, malignant, peritoneal or renal comorbidities) and/or the presence of large portosystemic collaterals on imaging studies are indicative of the presence of CSPH [3]. (A1)
6. Assessing the four Baveno stages of portal hypertension is clinically useful to quickly assess the prognosis of patients with liver cirrhosis: Baveno‑I compensated, no varices, Baveno‑II compensated, presence of GOVs, Baveno‑III decompensated with ascites and Baveno‑IV decompensated, history of variceal bleeding [3, 4]. (B1)

## II. Diagnosis and screening of portal hypertension

Fig. 1

**Fig. 1:**
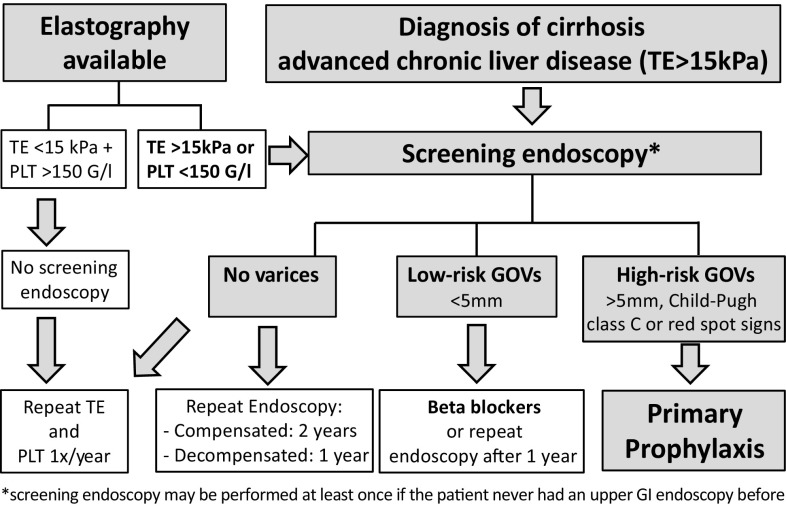
Flow chart for screening of varices in cirrhotic patients. *TE* transient elastography, *PLT* platelet count, *GOV* gastroesophageal varices

1. Patients with cirrhosis (or cACLD) should be screened for CSPH [3] (see Billroth-III screening algorithm in Fig. 1). (A2)
2. After the initial diagnosis of cirrhosis (or cACLD) screening endoscopy may be performed at least once if the patient never had an upper GI endoscopy before. (C1)
3. In cirrhotic patients with a platelet count >150 G/L and liver stiffness <15 kPa on transient elastography screening endoscopy can be safely deferred [5–7]. (B1)
4. Esophageal varices (EV) should be graded as absent, small (<5 mm of diameter), or large (≥5 mm). The presence of red spots should be indicated for risk stratification. (A2)
5. Gastric varices should be described as GOV-1 (continued varices on minor curvature), GOV-2 (continued varices on larger curvature extending to the fundus) or isolated gastric varices (IGV-1) isolated fundal varices or IGV-2 ectopic varices in the stomach. The presence of red spots should be indicated for risk stratification [8]. (B2)
6. In patients without varices, endoscopy should be repeated every 2 years in the case of compensated cirrhosis and every year in the case of decompensated cirrhosis [3]. (C1)
7. Patients with low-risk varices should receive non-selective beta blockers (NSBBs). (C1)
8. In compensated patients with varices (EV or GOV) receiving NSBBs there is no indication for endoscopic monitoring of the varices [3]. (C1)
9. If HVPG is measured as ≥10 mm Hg, endoscopy should be repeated every year in order to screen for the presence of varices, since CSPH is predictive of the formation of esophagogastric varices [3]. (A1)
10. There is no indication for subsequent endoscopic surveillance once large EVs or gastric varices (≥5 mm) are detected, unless endoscopic treatment is performed for primary or secondary prophylaxis of variceal bleeding [3]. (B1)

## III. Preprimary prophylaxis and prevention of decompensation

The effectiveness of NSBBs in the setting of preprimary prophylaxis (prevention of the development of varices and variceal bleeding in patients with compensated cirrhosis; cACLD) has been addressed in a landmark study which randomly assigned patients with cirrhosis and portal hypertension (defined by HVPG ≥6 mm Hg; 63% had CSPH) to timolol or placebo [9]. After a median follow-up of nearly 5 years, approximately 40% of patients in both groups met the composite primary endpoint of development of varices or variceal bleeding. Thus, in general, there is no indication for NSBBs treatment in patients who have not developed varices; however, NSBBs might be indicated for extrahepatic comorbidities (e. g. arterial hypertension, coronary heart disease, and heart failure). In the aforementioned study, patients who had a relative HVPG decrease of >10% after 1 year showed a lower incidence of the primary endpoint [9]; however, relevant HVPG decreases during NSBBs treatment are only observed in patients with CSPH [10]. In a recent randomized controlled trial (RCT) restricted to patients with CSPH, preprimary prophylaxis (44%) or small varices without red spot signs (56%), propranolol/carvedilol decreased the risk of hepatic decompensation, mostly by decreasing the incidence of ascites [11]. Thus, future studies should address the potential benefits of early initiation of NSBBs (especially carvedilol) treatment in the subgroup of patients with CSPH.

1. Preprimary prophylaxis defines the prevention of the development of varices and variceal bleeding in patients with compensated cirrhosis (cACLD) who do not have varices. (A1)
2. In general, there is no indication for NSBBs treatment in patients with cACLD who have not yet developed varices. Nevertheless, NSBBs might be indicated for extrahepatic comorbidities (e. g. arterial hypertension, coronary heart disease, and heart failure). (A1)
3. Preprimary prophylaxis with NSBBs can be considered in patients with CSPH since it may reduce the risk of developing ascites. (B2)

## IV. Primary prophylaxis of variceal bleeding

### Indications for primary prophylaxis

This chapter addresses primary prophylaxis in patients with esophageal varices (EV) and recommendations for the management of gastric varices is discussed in Chap. IX (gastric varices).

1. All patients with large EV (≥5 mm) should be treated either with NSBBs or with endoscopic variceal band ligation (EVL). The choice of treatment should be based on patient preference and characteristics as well as local resources and expertise. (A1)

### Choice of treatment for primary prophylaxis

2. Patients with small EVs with risk factors (red spot signs and/or with decompensated cirrhosis Child-Pugh class B or C) should receive NSBBs since they reduce the risk of bleeding in this setting [12]. (A1)
3. Patients with small EV without risk factors should also receive NSBBs prophylaxis, since NSBBs may reduce the incidence of variceal bleeding in this setting. (C1)
4. If monitoring of HVPG is available, treatment with NSBBs should be preferred, since achieving a hemodynamic response defines an excellent long-term prognosis [13]. (B1)
5. Hemodynamic response to NSBBs is defined as a reduction in HVPG ≤ 12 mm Hg or at least ≥10% from baseline. This is not only associated with a lower risk of first variceal bleeding but also with a lower incidence of ascites and death [14–16]. (A1)
6. The lack of access to HVPG measurement should not prevent physicians from using NSBBs for primary prophylaxis, since bleeding rates in primary prophylaxis are low even in hemodynamic non-responders to NSBBs. (B1)
7. Propranolol or carvedilol should be used for prophylactic pharmacological treatment of patients with varices. Carvedilol is more effective than propranolol in primary prophylaxis of variceal bleeding [17, 18]. (B1)
8. In patients with contraindications to NSBBs therapy, NSBBs intolerance, non-adherence to NSBBs or non-responders to NSBBs, EVL should be used. (B1)

### Endoscopic treatment

9. Use of EVL in primary prophylaxis should be performed in 2–6-week intervals until variceal eradication. A first follow-up endoscopy after variceal eradication should be performed after 6 months and then every 12 months. If EVL must be restarted the intervals are similar to first EVL [19]. (B1)

### Pharmacological treatment with NSBBs

10. There is no need for follow-up endoscopy in patients on pharmacological therapy. (B1)
11. The initial dose of propranolol is 20–40 mg twice daily with a maximum dosage of 160 mg/day in patients without and 80 mg/day with ascites. The initial dose of carvedilol is 6.25 mg once daily with a maximum dosage of 12.5 mg/day [16]. (B1)
12. The dose of NSBBs should be increased to achieve a resting heart rate of 55–60 beats per minute (bpm). The systolic blood pressure should not decrease below 90 mm Hg. (B1)
13. There is no relationship between reduction in portal pressure or protection from variceal bleeding and the reduction in resting heart rate or in blood pressure. There is no consensus on whether NSBBs treatment should be continued in patients without a hemodynamic response to NSBBs treatment; however, the benefit of NSBBs treatment may go beyond the portal pressure reducing effect and may also reduce the incidence of ascites, infections, decompensation and death [14, 15]. (B1)
14. In patients with severe or refractory ascites NSBBs should be discontinued during spontaneous bacterial peritonitis (SBP), a decline of systolic blood pressure <90 mm Hg or hyponatremia Na < 125 mmol/l or in the presence of acute kidney injury [20–22]. (C2)
15. Isosorbide mononitrate (ISMN) (alone or combined with NSBBs) is not recommended for primary prophylaxis, since it is not more effective in preventing first bleeding but increases side effects [23, 24]. (B1)
16. The combination of endoscopic treatment and NSBBs treatment does not further decrease the incidence of bleeding or death but is associated with a higher number of side effects and cannot be recommended for primary prophylaxis [25]. (A1)
17. The presence of varices does not represent an indication for proton pump inhibitors (PPIs); however, a short course of PPI post-variceal ligation reduces ulcer size and early bleeding risk [26, 27]. (C1)
18. Transjugular intrahepatic portosystemic shunt (TIPS) placement is not recommended for prevention of first variceal hemorrhage [28]. (C1)

## V. Acute variceal bleeding

**Fig. 2:**
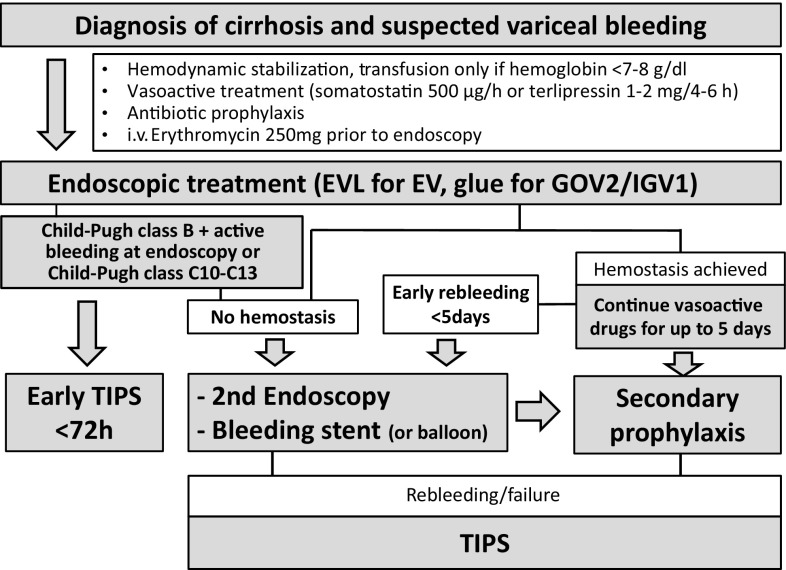
Flow chart for treatment of acute variceal bleeding. *EV* esophageal varices, *EVL* endoscopic variceal ligation, *TIPS* transjugular portosystemic shunt, *i.v.* intravenous

The Billroth‑III algorithm for treatment of acute variceal bleeding is summarized in Fig. 2

### Definition

1. Acute variceal bleeding (AVB) is diagnosed in cases of:
  - active bleeding at endoscopy or
  - signs of upper GI bleeding (hematemesis, blood or coagulated blood, melena) in patients with varices in the absence of any other source of bleeding.

### Blood products

2. Blood volume restitution should be done conservatively using packed red cells to maintain a Hb level of 7–8 g/dl (unless comorbidities/active bleeding necessitate more aggressive substitution), and substitution of fluids to maintain hemodynamic stability [29]. (A1)
3. Substitution of platelets may be considered if the platelet count is <50 G/L. (C2)
4. In the absence of disseminated intravascular coagulation (DIC), fibrinogen may be substituted if plasma levels are <100 mg/dL. (C2)
5. Correction of plasmatic coagulation indices cannot be generally recommended. (B1)

### Antibiotic prophylaxis

6. Antibiotic prophylaxis is an integral part of the therapy of variceal bleeding and should be started at admission with i. v. broad spectrum antibiotics which can be de-escalated according to culture results. In the absence of overt infections and successful control of AVB, antibiotic prophylaxis can be stopped after 5–7 days [30]. (A1)

### Vasoactive therapy

7. In case of suspected AVB vasoactive drugs should be started as soon as possible. (A1)
8. For vasoactive therapy, continuous i. v. somatostatin and terlipressin (administration as a bolus) have proven similar efficacy to control bleeding; however, terlipressin should be used with caution in patients with coronary artery disease (CAD), peripheral arterial occlusive disease (PAOD), cardiac arrhythmia, hyponatremia (<125 mmol/l), and severe asthma or chronic occlusive pulmonary disease (COPD). (A1)
9. Somatostatin: initially a bolus of 500 µg, afterwards 500 µg/h (6 mg/50 mL, 4.2 mL/h) by continuous infusion for up to 5 days.
10. Terlipressin: initially a bolus of 2 mg every 4 h. If the patient does not bleed for 24 h, bolus administration of 1 mg every 4 h should be continued for the next 24 h for up to 5 days. Continuous terlipressin infusion (initial dose of 2 mg/day; maximum 12 mg/day) can be used as well (see Chap. XII, HRS-AKI).
11. Vasoactive therapy may be maintained for up to 5 days to prevent early rebleeding. After this period, medicinal therapy for secondary prophylaxis should be started immediately. (A1)

### Prevention/therapy of hepatic encephalopathy

12. Lactulose or rifaximin can be used to prevent hepatic encephalopathy after AVB; however, more data on the risk-benefit ratio are needed. (B1)

### Prerequisites for facilities performing endoscopic therapy for AVB

13. Treatment of patients with AVB should be carried out by a GI endoscopist proficient in endoscopic hemostasis therapy together with support staff with technical expertise in the usage of endoscopic devices and treatment modalities, both with an availability on a 24 h-7 day basis. (B1)
14. Prerequisites for endoscopic therapy of AVB include (C1):
  Facilities for hemodynamic monitoring,
  Continuous monitoring of O_2_ saturation,
  Sufficient intravenous line for hemodynamic stabilization and treatment.
15. Intubation for endoscopy is desirable under one of the following conditions (C1):
  Massive and uncontrollable variceal bleeding,
  Hepatic encephalopathy (HE) grades III and IV,
  Risk of hypoxemia (failure to maintain blood oxygenation ≥90%),
  Evidence of aspiration.

### Endoscopic therapy

16. Endoscopic treatment should be performed as soon as possible after hemodynamic stabilization (at the latest 12 h after admission and ideally during the first 6 h), especially in patients with clinically significant bleeding or in patients with suspected cirrhosis. The therapeutic algorithm for AVB is summarized in Fig. 2. (C1)
17. Endoscopic treatment is best used in association with pharmacological therapy (vasoactive drugs + antibiotics) which should be started before endoscopy. (A1)
18. In the absence of contraindications, erythromycin improves visibility during endoscopy when administered 30–120 min before endoscopy. (A1)
19. Self-expanding metal stents are preferred to balloon tamponade as bridging to hemostatic therapy. (B1)
20. In AVB from EV endoscopic variceal ligation (EVL) is the preferred endoscopic therapy [31, 32]. (A1)
21. In AVB from cardiofundal varices (GOV2 and IGV1) injection of cyanoacrylate glue is the preferred endoscopic therapy. (A1)
22. In patients with signs of upper GI bleeding, endoscopic therapy of varices is highly recommended, even when no active bleeding can be detected by endoscopy. (B1)
23. Cyanoacrylate is not a standard treatment for EV but might be used as a rescue therapy of refractory bleeding. (C2)

### Prognosis

24. Active bleeding at endoscopy (under vasoactive therapy) is a poor prognostic sign regarding successful control of bleeding for the short-term period after variceal bleeding [33]. (B1)
25. An HVPG of ≥20 mm Hg, active bleeding at endoscopy and a Child-Pugh class C are associated with an increased failure to control bleeding and early mortality [34]. (B1)

### Failure to control bleeding

26. Failure to control bleeding (FCB) is defined as death or the occurence of one of the following complications within 5 days of the initial bleeding episode: (a) occurrence of fresh hematemesis, (b) development of hypovolemic shock or (c) drop in hemoglobin by ≥3 g/dl within any 24-h period as long as no blood transfusions are administered. (B1)

### Rebleeding

27. Clinically significant rebleeding is defined as recurrent melena or hematemesis resulting in hospital admission, blood transfusions, drop in Hb ≥ 3 g/dl, or death within 6 weeks after AVB. (B1)
28. Failure of secondary prophylaxis is defined as significant rebleeding related to portal hypertension occurring after AVB after initiation of secondary prophylaxis. (A1)

### Early transjugular intrahepatic portosystemic shunt (TIPS)

29. Early TIPS placement (within 72 h, ideally within 24 h) can prevent FCB; however, recent studies have shown conflicting results regarding mortality [35–37]. (B1)
30. Early TIPS placement should be performed in cases of AVB in the following scenarios: (i) in Child-Pugh class B patients with active bleeding at endoscopy despite vasoactive therapy, (ii) in all Child-Pugh class C patients with a Child score 10–13 and (iii) if HVPG is ≥20 mm Hg. (A1)
31. Contraindications for TIPS include: severe liver failure (Child-Pugh class > C13, Model for End-Stage Liver Disease [MELD] > 20), heart failure (in particular right heart failure), pulmonary hypertension, anatomical/technical contraindications, unrelieved biliary obstruction or extensive (hepatic) malignancy. (B1)
32. Acute HE at the time of AVB does not represent a contraindication for early TIPS. (C1)
33. Vasoactive drugs can be discontinued after successful TIPS placement. (C1)
34. Balloon occluded retrograde transvenous variceal obliteration (BRTO) may be considered in cases of (a) ongoing variceal bleeding after TIPS or (b) persistent large varices after TIPS. (C1)

## VI. Secondary prophylaxis of variceal bleeding

1. Secondary prophylaxis should be started as soon as vasoactive therapy is discontinued. (C1)
2. A combination of NSBBs and EVL represents the therapy of choice for secondary prophylaxis. (A2)
3. Endoscopic therapy of EVs in secondary prophylaxis must consider the presence of gastric varices. Usually GOV2/IGV1 should be treated prior to EVs. (C1)
4. Propranolol should be titrated to a daily dosage 80–160 mg/day (A2) or to a maximum of 80 mg/day in the presence of ascites. (C1)
5. Carvedilol (6.25–12.5 mg/day) is as effective as propranolol for lowering portal pressure in secondary prophylaxis (B2); however, in the presence of ascites carvedilol should not be used for secondary prophylaxis. (C1)
6. If there is new onset ascites while on NSBBs treatment, consider reducing the dose of propranolol and switch from carvedilol to propranolol. (C2)
7. Medical therapy with NSBBs alone is a valid choice for secondary prophylaxis if effectiveness can be documented by HVPG response by 20% decrease or to absolute HVPG values <12 mm Hg. (A2)
8. NSBBs non-responders in secondary prophylaxis require close EVL intervals (every 2–4 weeks) until variceal eradication. (A2)
9. EVL alone may be used for secondary prophylaxis in patients with contraindications to NSBBs. (A2)
10. ISMN monotherapy is not considered an alternative to NSBBs therapy; however, ISMN might be added to NSBBs in non-responders and HVPG-guided therapy would be preferable in this case. (C1)
11. EVL to prevent rebleeding in secondary prophylaxis should be continued at 2–4-week intervals until eradication of varices (small residual varices can be tolerated) and should then be repeated after 6 months and 12 months. If EVL must be restarted the intervals are similar to the first EVL.
12. Patients with advanced stage liver disease should be evaluated for liver transplantation. In these patients, endoscopic and/or medicinal therapy should be continued until liver transplantation. (C2)
13. EVL is the therapy of choice for variceal rebleeding (or insufficient decrease in HVPG on NSBBs), although EVL may have only moderate beneficial effects especially in these patients (B2).
14. TIPS is indicated in patients with failure of secondary prophylaxis and should be preferred over surgical shunts. (B1)
15. BRTO and surgical devasculariziation are a rescue therapy in patients with failure of secondary prophylaxis with NSBBs and EVL combination therapy if neither a TIPS nor shunt surgery is feasible. (C1)
16. TIPS should be considered for secondary prophylaxis in patients with severe/refractory concomitant ascites and/or in patients with NSBBs intolerance or non-response. (C1)
17. In patients with severe or refractory ascites NSBBs should be discontinued during SBP, a decline of systolic blood pressure <90 mm Hg or hyponatremia Na < 125 mmol/L or in cases of acute kidney injury (AKI).

## VII. Measurement of hepatic venous pressure gradient (HVPG)

1. Portal pressure, assessed by the hepatic venous pressure gradient (HVPG) drives the development of liver-related complications and mortality in patients with (compensated) advanced chronic liver disease (cACLD) [38, 39]. (A1)
2. HVPG measurements are indicated for assessing the prognosis and monitoring the response to etiologic and HVPG-lowering treatment [38, 39]. (A2)
3. The number needed to treat (NNT) for NSBBs for preventing variceal bleeding ranges from 5 (secondary prophylaxis) to 10 (primary prophylaxis) [40], underlining the need for methods to assess the expected benefits of NSBBs treatment in the individual patient [21]. (B2)
4. HVPG response is the only established surrogate for the effectiveness of NSBBs in preventing (recurrent) variceal bleeding. If HVPG decreases to a value of <12 mm Hg or is reduced by ≥20% during NSBBs treatment, patients are protected from variceal bleeding and survival is increased [41, 42]. (A1)
5. The assessment of acute HVPG response to intravenous propranolol (0.15 mg/kg given as 15 min infusion) provides a valuable alternative to chronic response assessment (separate measurements). An HVPG reduction by >10% or to <12 mm Hg (measured after the 15 min infusion) is sufficient in the acute setting [14, 43]. (A1)
6. Several studies support the use of HVPG-guided therapy. Thus, in centers with sufficient experience, HVPG response should be assessed to guide treatment decisions [11, 16, 44–47]. (A2)
7. HVPG measurements should be performed in fasting conditions. Since the procedure is generally well tolerated [48], ideally no sedation, or if necessary only low doses of midazolam (maximum 0.02 mg/kg) should be used [49, 50]. (A1)
8. HVPG measurements should be performed using a balloon catheter ensuring a sufficient wedge position and in order to maximize the assessed amount of liver parenchyma [51–53]. (A1)
9. Free hepatic venous pressure (FHVP) should be measured in a liver vein 2 cm from the inferior vena cava (stable values are usually obtained after 15 s) [54]. A difference between the inferior vena cava and FHVP > 2 mm Hg indicates misplacement or hepatic venous outflow obstruction [38]. (A1)
10. Wedged hepatic venous pressure (WHVP) should be measured after inflating the balloon of the catheter and verifying the wedge position by injecting contrast agent. Stable WHVP values may be expected only after at least 40 s [38]. (A1)
11. HVPG (FHVP subtracted from WHVP) is calculated as the mean of 3 measurements [38]. (A1)
12. For clinical study purposes, recording of the pressure tracings is mandatory [38]. (A1)

## VIII. Portal hypertensive gastropathy

**Fig. 3:**
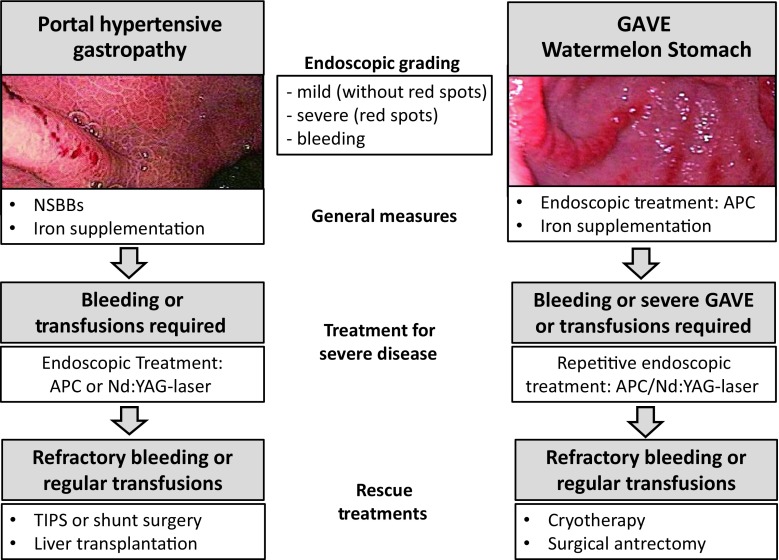
Flow chart for portal hypertensive gastropathy (PHG) and gastric antral vascular ectasia (GAVE). *APC* argon plasma coagulation, *GAVE* gastric antral vascular ectasia, *NSBBs* non-selective beta blockers, *PHG* portal hypertensive gastropathy, *TIPS* transjugular intrahepatic portosystemic shunt

1. Portal hypertensive gastropathy (PHG) is defined as a macroscopically visible mosaic-like pattern of the gastric mucosa (usually fundus or corpus) and can be found in 35–80% of cirrhotic patients, correlates with the Child-Pugh score and the degree of portal hypertension (PHT) [55]. A summary for the management of PHT is shown in Fig. 3. (A1)
2. PHG should be differentiated into mild PHG (without signs of bleeding) and severe PHG (red marks or active bleeding). (A1)
3. Gastric antral vascular ectasia (GAVE) is a distinct entity that is endoscopically characterized by tortuous columns of erythematous (mild) or hemorrhagic (severe) lesions in a “watermelon” or diffuse pattern (in the latter case histology may help to confirm diagnosis). GAVE may be present without cirrhosis and is associated with PHT in only 30% of cases [56]. (A1)
4. The incidence of acute PHG bleeding is 2–20% (mostly in severe PHG) [57]. (B2)
5. The incidence of chronic PHG bleeding is around 3–26% and is defined by a >2 g/dl decrease in Hb or by the presence of anemia together with positive faecal occult blood tests [57]. (B2)
6. If PHG is associated with iron deficiency anemia, iron substitution and in severe cases (Hb < 7 g/dL) transfusion should be considered. (B1)
7. There is no evidence for PHG screening or primary bleeding prophylaxis, yet the use of NSBBs for other indications is not discouraged. (C2)
8. Acute bleeding should be pharmacologically treated as AVB. Emergency gastroscopy should rule out other causes for GI bleeding and help to manage endoscopically treatable bleeding [58, 59]. (A1)
9. PHG with chronic bleeding should be treated with NSBBs [55, 57]. (B1)
10. In cases of refractory PHG bleeding TIPS, shunt surgery, argon-plasma coagulation (APC) or even liver transplantation represent rescue therapies. (B2)
11. GAVE bleeding should be treated by APC or Nd:YAG laser coagulation but multiple treatment sessions might be necessary [60]. (A1)
12. In severe or treatment resistant GAVE, band ligation, cryotherapy, radiofrequency ablation or surgical antrectomy represent potential salvage therapies [60]. (B2)
13. Pharmacotherapy or portocaval shunts do not play a role in the treatment of GAVE. (A1)

## IX. Gastric varices

1. The prevalence of gastroesophageal varices ranges between 17–20% in cirrhotic patients and may indicate the presence of portal or splenic vein thrombosis. (A2)
2. The Sarin classification should be used for classification of gastric varices: gastroesophageal varices type 1 and 2 (GOV1, GOV2) and isolated gastric varices 1 and 2 (IGV1, IGV2) [8]. (A1)
3. Risk of bleeding of gastric varices (1 year risk: 10–16%; 5 years risk: 44%) depends on subtype (IGV1 > GOV2 > GOV1), size, presence of red spots and the Child-Pugh score [61]. (A2)
4. GOV1 are considered extensions of EVs and should be managed similarly to EV in primary and secondary prophylaxis (including treatment with EVL). (C1) [62, 63].

### Primary prophylaxis for gastric varices

5. Primary prophylaxis of cardiofundal varices (GOV2 and IGV1) should be preferably performed with NSBBs [64]. (B1)
6. In patients with high-risk cardiofundal varices (≥10 mm) [64] elective cyanoacrylate glue injection may be considered for primary prophylaxis. (C2)
7. Neither TIPS, nor balloon-occluded retrograde transluminal oliteration (BRTO), or surgery are recommended for primary prophylaxis of gastric varices. (B1)

### Acute variceal bleeding from gastric varices

8. Initial management of patients with acute variceal bleeding from gastric varices is similar to bleeding from EVs, including vasoactive drugs, restrictive transfusion policy and antibiotic prophylaxis. (B1)
9. Cyanoacrylate glue injection is the treatment of choice for acute variceal bleeding from cardiofundal varices (GOV2, IGV1) and may be also used for GOV1 and IGV2 [63]. (A1)
10. A single injection should consist of maximum 1.0 ml of a cyanoacrylate/lipiodol mixture (1:1) in order to minimize the risk of embolization; however, more than one single injection is usually needed to obtain sufficient obliteration [65]. (B2)
11. Endoscopic variceal sclerotherapy is not recommended for treatment of acute or prophylactic treatment of gastric varices. (B1)
12. EVL is not an established therapy for bleeding GOV2, IGV1 + IGV2 due to a higher rebleeding rate compared to cyanoacrylate, EVL may only be performed on small GOV1 if technically feasible [62, 66]. (A2)
13. Early TIPS is indicated in high-risk patients with acute variceal bleeding from gastrofundal varices (GOV2, IGV1) as defined by (i) active bleeding at endoscopy, or (ii) Child-Pugh score C10–C13 or (iii) HVPG > 20 mm Hg [67]. (B1)
14. Linton-Nachlas balloon tamponade can be used as a bridge to hemostatic therapy in cases of failure to control bleeding from cardiofundal varices; however, risk of rebleeding after deflation is high. (B2)
15. BRTO represents an additional treatment option for bleeding cardiofundal varices. (B2)
16. Rarely surgical shunts, surgical devascularization (plus splenectomy), or splenic embolization are needed as rescue therapy for bleeding gastric varices not responding to vasoactive and endoscopic therapy if a TIPS cannot be performed. (C2)

### Secondary prophylaxis for gastric varices

17. Either NSBBs in combination with repeated cyanoacrylate glue applications in cases of high-risk cardiofundal varices (GOV1/GOV2/IGV1) or TIPS should be used for secondary prophylaxis after gastric variceal bleeding [62, 63]. (B1)
18. For secondary prophylaxis of small GOV1, EVL may be performed if technically feasible [62], in combination with NSBBs. (B2)
19. BRTO represents an additional treatment option for persistent cardiofundal varices, especially in patients with HE. (B2)
20. Rarely surgical shunts, surgical devascularization (plus splenectomy), or splenic embolization are needed as rescue therapy or in selected cases of left-sided portal hypertension (e. g. splenic vein thrombosis) if a TIPS cannot be performed. (C2)

## X. Management of ascites

30% of patients with compensated cirrhosis develop ascites within 5 years of follow-up [68]. Occurrence of ascites significantly impairs prognosis of liver cirrhosis, with a mortality of 15–20% within 1 year and 44% within 5 years [4, 69]. Treatment of ascites has not yet resulted in significant improvements in survival; however, treating ascites is important because it improves the quality of life of cirrhotic patients and the occurrence of SBP is unlikely in patients without ascites. Important definitions, grading and treatment are summarized in Table 2.

**Table 2:** Diagnosis and therapy of ascites

	Uncomplicated ascites			Refractory ascites
Definition	Grade 1: mild ascites only detectable by ultrasound	Grade 2: moderate ascites evident by moderate abdominal distension	Grade 3: large or gross ascites with marked abdominal distension	Ascites that cannot be mobilized or with early recurrence due to lack of response to sodium restriction and diuretic treatment; impaired urinary sodium excretion (<80 mmol/24 h); spot urinary sodium/potassium ratio <2.5
Treatment	Sodium restriction and diuretics		Paracentesis, sodium restriction and diuretics, Evaluation for OLT	TIPS or repetitive large volume paracentesis Liver transplantation must be considered
Avoid	NSAIDs, angiotensin converting enzyme inhibitors, angiotensin receptor blockers, aminoglycosides		NSAIDs, angiotensin converting enzyme inhibitors, angiotensin receptor blockers, aminoglycosides, carvedilol, propranolol with caution (not more than 80 mg/day)	

*NSAIDs* non-steroidal anti-inflammatory drugs, *TIPS* transjugular intrahepatic portosystemic shunt, *OLT* orthotopic liver transplantation

### Diagnostic approach in patients with ascites

1. Ascites should be graded according to the International Ascites Club guidelines into uncomplicated (grade 1: only visible on ultrasound, grade 2: moderate ascites, grade 3: massive ascites), and refractory ascites (not responsive or intolerant to diuretic therapy even after paracentesis) [70]. (A1)
2. Diagnostic paracentesis is indicated in (i) all cirrhotic patients presenting with ascites for the first time, (ii) cirrhotic patients with ascites with unscheduled admission to hospital regardless of the reason, and (iii) cirrhotic patients with ascites with signs of clinical deterioration (such as fever, hepatic encephalopathy, leucocytosis, abdominal pain, upper gastrointestinal bleeding or deterioration in renal function). Substitution of coagulation factors or platelets is not indicated even in patients with severe coagulopathy, because paracentesis rarely leads to serious bleeding complications [71, 72]. (B1)
3. Investigation of ascites should include at least determination of ascitic neutrophil count, protein concentration, and the serum-ascites albumin gradient (SAAG). Uncomplicated ascites due to portal hypertension is expected to show a neutrophil count <250/µl, a SAAG >1.1 g/dl [73] and a protein level <2.5 g/dl. The SAAG is calculated by subtracting the ascitic fluid albumin level from the serum albumin level (both determined on the same day). (B1)
4. Additionally, aerobic and anaerobic blood culture bottles should be inoculated with ascitic fluid for bacteriological diagnosis of SBP or bacterascites (neutrophil count <250/µl but positive ascites fluid culture). (B1)

### Therapy of uncomplicated ascites

5. Initial therapy of patients with cirrhosis and ascites consists of moderate sodium restriction (90 mmol NaCl/day, corresponding to 5.2 g NaCl/day), and diuretic therapy. Sodium restriction to less than 5 g NaCl/day is not recommended due to the risk of aggravating malnutrition that is usually present in these patients [74]. (B1)
6. Diuretic therapy should be started with spironolactone 100 mg and furosemide 40 mg [75, 76]. In the case of insufficient ascites control or lack of effectiveness, doses of spironolactone and furosemide can be increased by 100 mg and 40 mg every 3–5 days. The daily dose of 400 mg spironolactone and 160 mg furosemide should not be exceeded. (A1)
7. Furosemide should not be administered intravenously as a bolus in cirrhotic patients because of risk of deterioration in the glomerular filtration rate (GFR) [77]. (B1)
8. The use of spironolactone or amiloride as single agents or combined with thiazides may have a role for outpatients or previously untreated patients due to a lesser need for dose adjustments [78, 79] (B1)
9. Eplerenone is an alternative for men with gynecomastia, but has not been compared to spironolactone or furosemide in the setting of portal hypertensive ascites [80]. 100 mg of spironolactone is considered equivalent to 50 mg of eplerenone. Furthermore, amiloride as single agent or combined with thiazides may have a role in patients who are intolerant or develop side effects to spironolactone or furosemide [81]. (B2)
10. Vaptans are not beneficial for the long-term management of portal hypertensive ascites [82]. (A1)
11. Rapid weight loss during diuretic therapy might increase the risk of hypovolemia, AKI and hepatic encephalopathy and thus, weight loss during diuretic therapy should not exceed 1 kg/day or 4 kg/week. (B2)
12. In patients with tense ascites (grade 3), paracentesis is the treatment of choice and should be followed by diuretic therapy. Total paracentesis should be carried out as a single procedure, even when a large volume of ascites is present, as long as it is hemodynamically tolerated by the patient. (B1)
13. Plasma volume expansion using albumin is recommended in all patients undergoing paracentesis if more than 5 l of ascites have been removed, for prevention of hypovolemia and circulatory dysfunction [83]. Albumin at a dose of 8 g/l of ascites removed should be administered (i. e. 100ml 20% albumin per 2.5 l ascites removed). Removal of less than 5 l does not appear to have hemodynamic consequences [84]. (A1)
14. Patients responsive to diuretics should primarily be treated with sodium restriction and diuretics and should not undergo serial paracentesis. (B1)
15. In cirrhotic patients with severe hyponatremia (plasma sodium levels <125 mmol/l) fluid restriction is recommended since the underlying pathophysiology is usually dilutional/hypervolemic hyponatremia. (A1)
16. In severe hyponatremia diuretics should be stopped, since at these levels diuretics are ineffective and worsen hyponatremia. Substitution with concentrated NaCl solutions should be avoided [85]. (C2)
17. If hyponatremia occurs together with hepatic encephalopathy or with AKI, plasma volume expansion with saline and/or albumin should be considered. (C2)
18. Patients with moderate to severe ascites should be evaluated for liver transplantation. (B1)
19. The administration of non-steroidal anti-inflammatory drugs (NSAIDs) in patients with decompensated cirrhosis and ascites can lead to renal failure and therefore should be avoided [86]. The same is true for angiotensin receptor blockers and angiotensin converting enzyme inhibitors [87, 88]. Aminoglycosides should only be used in cases where infections cannot be otherwise treated [89, 90]. (A1)
20. In the absence of strong indications, proton pump inhibitors (PPIs) should not be used in patients with ascites since PPIs might be associated with a higher risk of infections [91]. (A2)
21. Ascites per se is not a contraindication for NSBBs, but they should be used with caution. Carvedilol should not be used in patients with severe or refractory ascites due to induction of hypotension [92]. In patients with severe or refractory ascites, high doses of propranolol (>80 mg/day) should be avoided [93]. (C2)

### Refractory ascites

Only less than 10% of patients with cirrhosis and ascites are refractory to treatment regimens consisting of sodium restriction and oral diuretics [94].

22. Refractory ascites is defined by the International Ascites Club [70] (A1):
  as ascites that cannot be mobilized by intensive diuretic therapy (up to a maximum of 400 mg spironolactone and 160 mg furosemide per day) and confirmed dietary sodium restriction (diuretic-resistant ascites),
  or as ascites that rapidly reaccumulates after therapeutic paracentesis (within 4 weeks),
  or as the situation, where the maximum dose of diuretics cannot be administered due to side effects, such as electrolyte imbalance, renal failure, and encephalopathy (diuretic-intolerant ascites).
23. Refractory ascites can develop secondary to hepatocellular carcinoma or portal vein thrombosis; therefore, ultrasound examination should be performed to exclude these complications of cirrhosis. (B1)
24. A characteristic feature of refractory ascites is impaired urinary sodium excretion despite maximum tolerated doses of diuretics [95]. Since urine collection for 24 h is cumbersome, a spot urinary sodium/potassium ratio <2.5 is a reasonable surrogate for diuretic-resistant ascites [96]. Diuretic treatment should be continued only when urinary sodium excretion under diuretic therapy is greater than 30 mmol/day [97]. (B2)
25. Due to the poor prognosis of patients with refractory ascites liver transplantation must be considered. (A1)
26. Patients with refractory ascites should be evaluated for TIPS, since TIPS is associated with improved survival [98–101]. (A1)
27. If TIPS is contraindicated or refused by the patient, repetitive large volume paracentesis in combination with albumin substitution, sodium restriction and diuretic therapy should be performed. (B1)
28. The efficacy and safety of low-flow pump systems to remove ascites from the peritoneal cavity into the bladder in patients with refractory ascites remains to be established [102, 103]. (C2)
29. In patients with severe/refractory ascites NSBBs should be discontinued during SBP [20], a decline of systolic blood pressure <90 mmHg, hyponatremia <125 mmol/L or in the presence of AKI. (C1)

### Hepatic hydrothorax

30. Hepatic hydrothorax represents a (usually a right-sided) pleural effusion in patients with cirrhosis and ascites in the absence of any other pleural or pulmonary disease [104]. (A1)
31. Diagnostic pleuracentesis of hepatic hydrothorax should be performed at first diagnosis and include similar testing as for ascitic fluid. (B1)
32. The absolute neutrophil count is usually higher than in the ascitic fluid and thus, the diagnosis of bacterial infection of the pleural effusion should mainly be based on culture results [105]. (C2)
33. Hepatic hydrothorax should be primarily treated with salt restriction and diuretics [106]. (B1)
34. TIPS should be considered for recurrent hepatic hydrothorax not responsive to diuretic therapy [107, 108]. (B1)
35. Other treatment modalities including pleurodesis [109] or permanent drainage systems [110] cannot be recommended for treatment of hepatic hydrothorax. The role of novel indwelling pleural catheters is not yet clear [111]. (B2)
36. Patients with recurrent hepatic hydrothorax should be evaluated for liver transplantation [112]. (A1)

## XI. Spontaneous bacterial peritonitis (SBP)

1. All patients presenting with ascites for the first time, with recurrence of ascites, or deterioration of ascites, evidence of systemic infection, GI bleeding, worsening liver or renal function, or hepatic encephalopathy should undergo paracentesis to screen for SBP [97]. (A1)
2. Ascitic fluid and blood cultures should be performed using blood culture bottles. Even in culture-negative SBP, positive blood cultures might hint at the responsible organism [97]. (A1)
3. In patients with an ascitic fluid absolute neutrophil count >250/µl or a positive ascitic fluid culture, antibiotic therapy with gram-negative coverage (e. g. aminopenicillin/beta-lactamase inhibitor, third generation cephalosporin, or quinolone) should be started immediately. (A1)
4. Chinolones should not be used to treat SBP in patients who were on norfloxacin prophylaxis [97]. (B1)
5. In selected high-risk patients (e. g. nosocomial SBP as defined by onset of signs and symptoms of infection after 72 h from hospitalization and/or patients with sepsis), the use of combination regimens as initial therapy might be warranted [113]. (A2)
6. To prevent the development of hepatorenal syndrome (HRS) type of AKI, 1.5 g/kg bodyweight albumin should be administered in patients with SBP at the time of diagnosis, plus 1 g/kg body weight on day three [114]. (A1)
7. Blood pressure should be carefully monitored in patients with SBP and NSBBs should be discontinued in the case of systolic blood pressure <90 mm Hg, hyponatremia Na < 125 mmol/L, or AKI [21, 22]. (C2)
8. In the case of an ascitic fluid neutrophil count <250/µL but clinical evidence of infection, similar antibiotic therapy should be initiated and continued until culture results are available [97]. (B1)
9. A second paracentesis should be performed 48 h after initiation of the antibiotic therapy to demonstrate a decrease of the ascitic absolute neutrophil count by 25% of the initial value [115]. (A1)
10. A smaller drop is highly suggestive of failure of the antibiotic regimen. In these patients, antibiotic therapy should be adopted based on culture results and susceptibility testing [97]. (A1)
11. If culture-negative, antibiotic therapy should be changed to cover gaps in the antibacterial spectrum of the initial therapy, as well as relevant multidrug-resistant gram-negative and gram-positive bacteria (e. g. meropenem plus daptomycin) [113]. (B1)
12. Due to the poor prognosis of patients who recovered from SBP, liver transplantation should be considered in these patients [97]. (A1)
13. All patients with a history of SPB should be treated continuously with secondary prophylaxis using norfloxacin 400 mg/day or alternatively co-trimoxazole (800 mg/160 mg/day) [97]. (A1)
14. Given the inevitable risk of antibiotic resistance, the use of prophylactic antibiotics in patients without a history of SBP should be restricted to patients at high risk for SBP: low ascites protein (<15 g/l) with advanced liver failure (Child-Pugh score ≥9 points with serum bilirubin ≥3 mg/dL) or impaired renal function (serum creatinine sCr ≥ 1.2 mg/dL, blood urea nitrogen ≥25 mg/dl, or serum sodium ≤130 mmol/L) [116, 117]. (C1)
15. In patients with Child-Pugh C10-15 norfloxacin prophylaxis seems to decrease 6-months mortality. (B1) [118]
16. Based on the currently available evidence, rifaximin cannot be used as a substitute for norfloxacin/co-trimoxazole [119–124]. (C1)
17. In patients diagnosed with SBP while on norfloxacin prophylaxis, secondary prophylaxis should be chosen on an individual basis considering culture results and susceptibility testing. (C2)
18. If a patient on prophylactic antibiotics develops other recurrent infections (e. g. cholangitis or urinary tract infections), antibiotics with a higher oral bioavailability than norfloxacin should be used. (C2)

## XII. Management of acute kidney injury and hepatorenal syndrome (HRS-AKI)

Acute kidney injury (AKI) is a common complication of cirrhosis with a significant prognostic impact [125, 126]. As a consequence of systemic and splanchnic arterial vasodilatation, renal perfusion is critical in patients with advanced cirrhosis and CSPH [127]. AKI is commonly triggered by precipitating events leading to further circulatory compromise including overdose of diuretics, large volume paracentesis without albumin replacement, GI blood loss, and infections (e. g. SBP) [128].

### Diagnosis and definitions

The traditional diagnostic criteria of renal failure in cirrhosis (percentage increase in sCr, ≥50% to a final value ≥1.5 mg/dl) [129] were replaced by the Kidney Disease Improving Global Outcome (KDIGO) criteria to diagnose AKI [130] and adapted for patients with cirrhosis by the International Club of Ascites (ICA) in 2015 [131]. One of the main modifications of the ICA-AKI criteria is the abandonment of a threshold of sCr ≥ 1.5 mg/dl to diagnose AKI in cirrhosis, since smaller rises in sCr have also been shown to have a negative prognostic impact in these patients [126, 132].

**Fig. 4:**
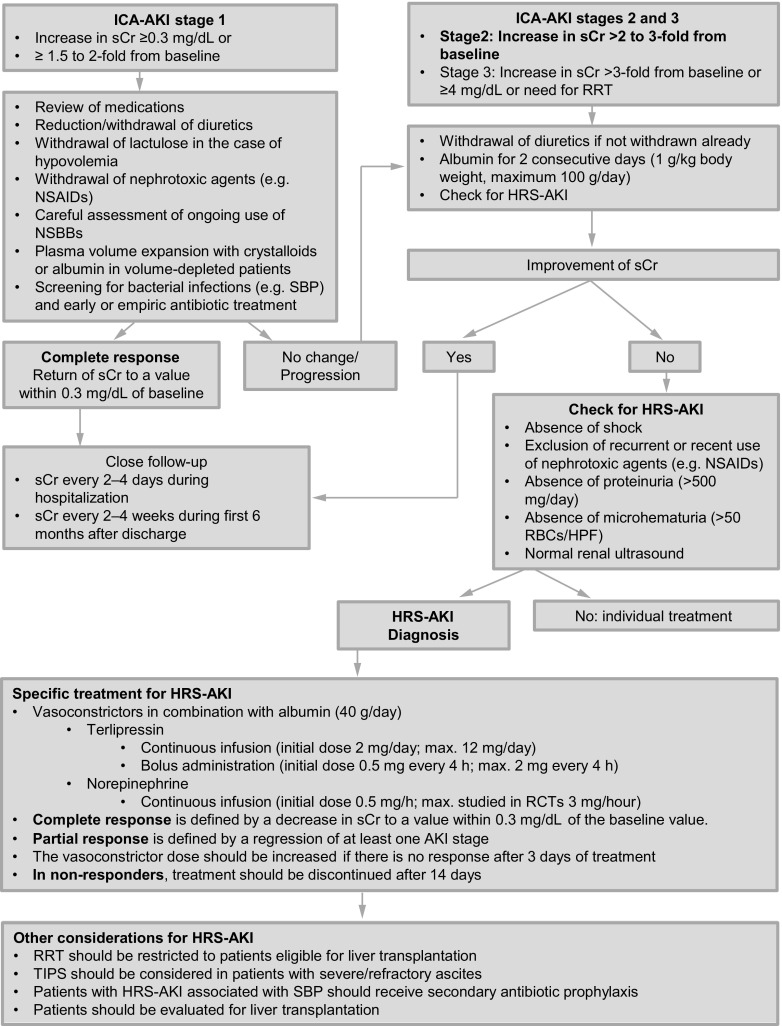
Management of AKI in cirrhosis. Adapted from [133] (*AKI* acute kidney injury, *ICA* International Club of Ascites, *HPF* high power field, *HRS* hepatorenal syndrome, *NSAIDs* non-steroidal anti-inflammatory drugs, *NSBBs* non-selective beta blockers, *RBCs* red blood cells, *RRT* renal replacement therapy, *SBP* spontaneous bacterial peritonitis, *sCr* serum creatinine)

A detailed algorithm for diagnosis and treatment of AKI in patients with cirrhosis is shown in Fig. 4.

### Diagnosis and definitions

1. AKI in cirrhosis should be diagnosed according to the ICA-AKI criteria [131]. (B1):
  Increase in sCr ≥ 0.3 mg/dl within 48 h or
  Increase in sCr ≥ 50% from a baseline value that is known or presumed to have occurred in the past 7 days.
  A baseline sCr value obtained in the previous 3 months should be used. If no previous sCr value is available, the sCr on admission should be used. In cases of impairment of renal function (sCr ≥ 1.5 mg/dl) at time of admission and a clearly identifiable precipitating event, it is reasonable to assume AKI based on clinical judgement.
  The use of a reduction in urine output as part of the diagnostic criteria was eliminated in the new ICA criteria for the diagnosis of AKI because many patients with cirrhosis and ascites are oliguric as part of the sodium and water retention syndrome and yet maintain a nearly normal GFR [131, 134]. Based on that only the changes in sCr should be used to diagnose AKI in patients with cirrhosis (B1).
2. AKI in cirrhosis should be staged according to the ICA-AKI criteria [131]*: (B1)*
  ICA-AKI stage 1: increase in sCr ≥ 0.3 mg/dl or ≥1.5 to 2‑fold from baseline
  ICA-AKI stage 2: increase in sCr > 2 to 3‑fold from baseline
  ICA-AKI stage 3: increase in sCr > 3‑fold from baseline or ≥4 mg/dl with an acute increase ≥0.3 mg/dl or need for renal replacement therapy (RRT)
3. The hepatorenal syndrome type of AKI (HRS-AKI, formerly known as HRS type 1) is defined as ≥ stage 2 ICA-AKI fulfilling all other diagnostic criteria of HRS-AKI [131]*: *(A1)
  Presence of ascites
  No improvement in sCr after 2 consecutive days of withdrawal of diuretics and plasma volume expansion with albumin (1 g/kg, max.100 g/day)
  Absence of shock
  Exclusion of nephrotoxic agents (e. g. NSAIDs, aminoglycosides, contrast media)
  Exclusion of parenchymal kidney disease (proteinuria <500 mg/day, <50 red blood cells per high power field, normal renal ultrasound)
4. Hepatorenal syndrome type 2 is defined as slowly progressive impairment of renal function (sCr > 1.5 mg/dl) [135, 136] fulfilling the abovementioned diagnostic criteria of HRS-AKI and is usually associated with refractory ascites [125, 126] (A1).

### Management of AKI and HRS-AKI in cirrhosis

The initial management of AKI should focus on identification and correction of precipitating factors that further exaggerate the already disturbed hemodynamics in advanced cirrhosis [131, 137, 138].

5. The following measures should be taken in cirrhotic patients with initial ICA-AKI stage 1. (A1)
  Review of all medications (including over the counter drugs)
  Reduction or withdrawal of diuretic therapy and/or lactulose for patients who are volume-depleted from diuretics or excess lactulose use
  Withdrawal of all potentially nephrotoxic agents (e. g. NSAIDs)
  Careful assessment of ongoing use of drugs potentially inducing/aggravating hypotension (e. g. NSBBs) [93, 139]
  Plasma volume expansion with crystalloids or albumin in patients with clinically suspected hypovolemia
  Blood transfusion in patients with AKI after GI blood loss
  Screening for bacterial infections (e. g. SBP) and early or empiric antibiotic treatment if an infection is diagnosed or strongly suspected [140]
6. In the case of response (return of sCr to a value within 0.3 mg/dl of the baseline value), patients should be followed closely for early identification of potential new episodes of AKI [131, 141]. (B2)
  Assessment of sCr every 2–4 days during hospitalization
  Assessment of sCr every 2–4 weeks during the first 6 months after discharge
7. In the case of stage 2 or 3 ICA-AKI or progression of stage 1 ICA-AKI to a higher stage, patients need to be assessed for the presence of HRS-AKI in addition to the following measures[131]. (B1):
  Administration of the same general measures as described for patients with ICA-AKI stage 1,
  Withdrawal of diuretics if not withdrawn already,
  Plasma volume expansion with albumin for two consecutive days (1 g/kg body weight, maximum 100 g/day).

### Treatment of HRS-AKI

8. Patients with HRS-AKI should be treated with vasoconstrictors (terlipressin or norepinephrine) in combination with albumin (40 g/day) [131]. (A1).
9. Patients with ICA-AKI stage 1 and sCr < 1.5 mg/dl fulfilling the diagnostic criteria of HRS-AKI can be treated the same way on a case-by-case basis [131]. (C2).
10. Patients with HRS type 2 can be treated similarly [142–144]. (A1).

### Vasoconstrictor treatment

11. Patients receiving vasoconstrictors should be preferably treated in an intermediate care (IMCU) or intensive care unit (ICU). (B1)
12. Vasoconstrictors should be preferably administered via a central venous line under continuous blood pressure and electrocardiography (ECG) monitoring. (B1)
13. Non-availability of an IMCU/ICU should, however, not defer the use of vasoconstrictors in patients with HRS-AKI. (B1)

### Terlipressin

14. Terlipressin is the most intensively studied vasoconstrictor for the treatment of HRS-AKI. (A1)
15. A bolus of terlipressin induces a statistically significant reduction in portal pressure over 3–4 h and also increases mean arterial pressure [145]. (A1).
16. Terlipressin should be used with caution in patients with cardiovascular disease, since it may induce ischemia. (A1)
17. Patients should be monitored for hyponatremia, which more commonly occurs in patients with less advanced liver disease and (near) normal baseline serum sodium levels [146]. (A1).
18. Continuous infusion (initial dose of 2 mg/day; maximum 12 mg/day) decreases the rate of adverse events, the mean effective terlipressin dose and, thus, might also decrease costs as compared to bolus administration (initial dose of 0.5 mg every 4 h; maximum 2 mg every 4 h). Continuous infusion might be preferred over bolus administration [147]. (A1)
19. Although terlipressin has been consistently shown to improve renal function, its impact on survival is less clear [148]. (A1)
20. Terlipressin is particularly beneficial in patients with systemic inflammatory response or sepsis and might also prevent variceal bleeding during the period of discontinuation of NSBBs [149]. (B2)

### Norepinephrine

21. Norepinephrine (initial dose of 0.5 mg/h; maximum dose studied in RCTs: 3 mg/h) is an equally effective and inexpensive alternative to terlipressin [150]. (A1)

### Response to treatment and considerations for follow-up

22. Complete response to treatment is defined by a decrease in sCr to a value within 0.3 mg/dl of the baseline value, while a regression of at least one AKI stage is considered as partial response. (B1)
23. The vasoconstrictor dose should be increased (terlipressin continuous infusion: maximum 12 mg/day; bolus administration: maximum 2 mg every 4 h; norepinephrine: maximum dose studied in RCTs 3 mg/h), if there is no response after 3 days of treatment. (A1)
24. In non-responders, treatment should be discontinued after 14 days. (B1)
25. In responders, longer treatment durations can be used as a bridging therapy prior to liver transplantation. (B1)
26. Recurrent HRS-AKI should be treated in the same way. (A1)
27. HRS type 2 commonly recurs after cessation of vasoconstrictor treatment. There is no evidence for beneficial effects of vasoconstrictor treatment on pre-transplantation and post-transplantion outcomes [144, 149, 151]. (A1)

### Other treatment considerations for AKI and HRS-AKI

28. TIPS might improve kidney function in patients with HRS-AKI. Additional indications for TIPS placement might be present in a relevant proportion of patients with HRS-AKI [152–155]. (A1)
29. Patients with HRS type 2 should be evaluated for TIPS, since TIPS improves both renal function and survival in patients with severe/refractory ascites [98, 156]. (B1)
30. Since TIPS can deteriorate liver function, serum bilirubin >5 mg/dl represents a contraindication for TIPS implantation for the treatment of HRS type 2 and HRS-AKI [157]. (A1) (see also Chap. 13, TIPS).
31. There are no RCTs demonstrating that renal replacement therapy (RRT) or extracorporeal liver support (ELS) improves survival in patients with HRS-AKI and HRS type 2, or associated conditions, such as acute-on-chronic liver failure (ACLF) [158, 159]. (A1)
32. RRT and ELS should be restricted to patients who are eligible for liver transplantation; however, even in this setting, there is no evidence of a survival benefit. (B1)
33. In absence of head-to-head comparisons, the optimal modality of RRT is unclear; however, continuous RRT may be advantageous in patients who are hemodynamically unstable or at risk of elevated intracranial pressure (e. g. ACLF) [160]. (B1)
34. Patients with HRS-AKI or HRS type 2 should be evaluated for liver transplantation. (A1)
35. Although HRS-AKI and HRS type 2 usually resolve completely after liver transplantation, combined liver and kidney transplantation should be considered in patients on RRT for more than 12 weeks. (C2)
36. Albumin should be administered in all large volume paracenteses (>5 L), since it prevents postparacentesis circulatory dysfunction, and thus HRS-AKI, and might even improve survival [83, 161]. (A1)
37. Norfloxacin treatment (400 mg/day) prevents SBP and therefore HRS-AKI development in selected patients with ascites. (See Chap. XI, SBP). (A1)

## XIII. Transjugular intrahepatic portosystemic shunt (TIPS)

1. Dedicated polytetrafluoroethylene (PTFE)-covered stents are superior to bare metal stents for TIPS [162–165]. (A1)

### TIPS for variceal bleeding

2. All bleeding indications for TIPS apply for patients with EV and gastric varices. (B1)
3. Early TIPS (<72 h) for acute variceal bleeding should be placed (i) in all patients with Child-Pugh class C10–C13, (ii) in patients with Child-Pugh class B and active bleeding at endoscopy or (iii) if HVPG is ≥20 mm Hg [36, 37, 166]. (B1)
4. Early TIPS should not be used in patients with Child-Pugh class C14–C15, MELD > 20, extensive hepatic malignancy, or severe renal insufficiency (creatinine >3 mg/dL). (A1)
5. Rescue TIPS should be used in patients with refractory/uncontrollable variceal bleeding (rebleeding under continued vasoactive therapy or after placement of an esophageal bleeding stent) [167]. (A1)
6. Elective TIPS for secondary prophylaxis of variceal bleeding should be considered in patients with (i) failure of NSBBs+EVL, (ii) intolerance to NSBBs, (iii) in the case of concomitant ascites, or (iv) in patients with cardiofundal varices (GOV2/IGV1) [47]. (B1)
7. Contraindications for TIPS include: severe liver failure (Child-Pugh class > C13, MELD > 20), heart failure (in particular right heart failure), pulmonary hypertension, anatomical/technical contraindications, unrelieved biliary obstruction or extensive (hepatic) malignancy. (A1)
8. Acute HE at time of AVB does not represent a contraindication for a bleeding TIPS. (B2)
9. In case of persistent bleeding after TIPS variceal embolization should be performed (A2); BRTO may be considered in selected cases [168–170]. (B2)
10. Adjunctive variceal embolization during TIPS may be considered in patients with contrast material filling of varices after shunt creation. (C2)
11. TIPS is not recommended for prevention of first variceal hemorrhage. (A1)
12. After TIPS, vasoactive drugs can be discontinued and patients do not require NSBBs or EBL. (C1)
13. Patients with TIPS should be evaluated for liver transplantation. (A1)

### TIPS for refractory ascites

14. Diagnosis of refractory portal hypertensive ascites must be ascertained before evaluating patients for TIPS. (A1)
15. Diuretic-refractory ascites is defined as recurrent ascites despite 400 mg spironolactone and 160 mg of furosemide while on dietary sodium restriction to 5.2 g/day. (A1)
16. Diuretic-intolerant ascites is defined as recurrent ascites due to intolerance/side effects to maximum dose of spironolactone/furosemide. (A1)
17. In the absence of contraindications TIPS represents the treatment of choice for refractory ascites, since TIPS increases survival as compared to repetitive larve volume paracentesis (LVP) plus albumin [101, 156]. (A1)
18. Recurrent spontaneous HE episodes in the absence of triggers, such as bleeding, infections, electrolyte disturbances and overdose of diuretics are a contraindication against TIPS. (C1)
19. In patients with refractory ascites, a bilirubin >5 mg/dL and severe renal failure (sCr >3 mg/dL) represent contraindications against TIPS. (A1)
20. Further contraindications for TIPS include: severe liver failure (Child-Pugh class > C13, MELD > 20), heart failure (in particular right heart failure), pulmonary hypertension, anatomical/technical contraindications, or extensive (hepatic) malignancy. (A1)
21. Patients undergoing TIPS implantation should receive medication HE prophylaxis (rifaximin or lactulose or iv. L‑ornithin L‑asparate), which can be discontinued later depending on the clinical presentation of the patient. (B2)
22. Resolution of ascites after TIPS is slow and most patients require continued administration of diuretics and fluid restriction afterwards. (B1)
23. Anticoagulation and anti-platelet drugs are not mandatory after TIPS implantation. (C1)

### TIPS for other indications

24. Patients with severe/refractory hepatic hydrothorax may be treated with TIPS. (C1)
25. TIPS represents a therapeutic option for patients with HRS type 2. (B1)
26. TIPS represents a rescue therapy for bleeding from PHG if vasoactive drugs fail. (B1)
27. TIPS or angioplasty (sometimes in combination with stents) should be used in patients with Budd-Chiari Syndrome (BCS) who do not improve under anticoagulation therapy [171–174]. (B1)
28. In patients with BCS without cirrhosis hyperbilirubinemia >5 mg/dL is not a contraindication against TIPS implantation [175]. (C1)
29. TIPS (in combination with anticoagulation) should be considered for acute non-malignant portal vein thrombosis (PVT) in symptomatic patients (i. e. ascites and/or risk of intestinal infarction) in order to perform clot removal [176]. (B1)
30. Anticoagulation after TIPS is not necessary in all patients with PVT, but should be used in patients with a persistent prothrombotic condition and in BCS patients [172, 177]. (B1)
31. TIPS is indicated in patients with severe non-cirrhotic portal hypertension (NCPH), a syndrome that includes idiopathic NCPH (INCPH), sinusoidal obstruction syndrome (SOS, previously termed veno-occlusive disease, VOD), sarcoidosis, congenital fibrosis and portal sclerosis. (B1)

### Evaluation of patients with portal hypertension for TIPS

32. Interdisciplinary boards involving hepatologists and interventional radiologists should be implemented to decide on TIPS implantation. (C1)
33. Presence and etiology of portal hypertension must be confirmed prior to TIPS. (A1)
34. Indications for TIPS implantation include control of acute variceal bleeding (early TIPS, rescue TIPS) and prevention of variceal rebleeding (elective TIPS for secondary prophylaxis) and refractory ascites. (A1)
35. Contraindications for TIPS include: severe liver failure (Child-Pugh class > C13, MELD > 20), heart failure (in particular right heart failure), pulmonary hypertension, anatomical/technical contraindications, unrelieved biliary obstruction, history of recurrent spontaneous episodes of HE West Haven grades III/IV, or extensive (hepatic) malignancy. (B1)
36. Evaluation for TIPS must include a sufficient imaging study of portal and hepatic veins, e.g. Doppler ultrasound (DUS), computed tomography (CT) and magnetic resonance imaging (MRI) (A1)
37. Evaluation for TIPS should include echocardiography to exclude heart failure (especially right heart failure) and to estimate systolic pulmonary arterial pressure (sysPAP) to exclude pulmonary hypertension. (B1)
38. Evaluation for TIPS should exclude hepatic insufficiency. Thus, Child-Pugh and MELD scores should be calculated. (A1)
39. In the case of ascites, paracentesis prior to TIPS should be performed to exclude malignant ascites and SBP, and to improve technical performance of TIPS procedure. (B1)

### TIPS procedure

40. Dedicated PTFE-covered endoprotheses should be used and implanted with an adequate extent into the hepatic vein for TIPS creation. (A1)
41. The procedure should be performed with the patient under sedoanalgesia or general anesthesia. (B1)
42. Antibiotic prophylaxis (e. g. cephalosporins) should be considered during TIPS procedures. (C2)
43. Digital subtraction angiography equipment with high-quality fluoroscopy with zooming and reference imaging should be available. Puncture of the portal vein can be navigated by ultrasound guidance or carbon dioxide wedged hepatic venography to identify the portal vein. (C1)
44. Endoprotheses with 10 mm nominal diameter with primary dilation to 8 mm are recommended. (B1)
45. Portosystemic pressure gradient (PPG: portal venous pressure, IVC/RA) should be calculated prior to and after TIPS implantation. (A1)
46. PPG should be aimed at <12 mm Hg and >8 mm (at minimum a decrease of >50% in patients with high PPG > 30 mm Hg prior to TIPS should be achieved); PPG must not be within the range of normal portal pressure. (B1)
47. The impact of additional variceal embolization is not validated; however, embolization of persisting large portosystemic shunts may be performed in order to decrease the risk of overt HE. (C2)
48. Intraprocedural application of heparin should be considered with doses adjusted to coagulation status and TIPS indication. (C2)

### Care after TIPS implantation

49. Anticoagulation and anti-platelet drugs are not mandatory after TIPS implantation. (B1)
50. DUS is recommended 3–5 days after TIPS and every 6 months thereafter. (B1)
51. If shunt dysfunction is suspected, portography and pressure measurements are indicated and if verified, revision should be performed to avoid clinical deterioration. (B1)
52. In patients with poor clinical response (evaluated after at least 3 months after TIPS implantation) portography and PPG measurement are recommended.
53. In the case of recurrent spontaneous or persistent HE episodes West Haven grade III/IV, a reduction of TIPS diameter should be performed. (A1)

## XIV. Portal vein thrombosis (PVT)

1. Characterization of PVT should distinguish: (a) acute from chronic PVT, (b) obstructive from non-obstructive PVT, (c) malignant vs. non-malignant PVT and (e) cirrhotic vs. non-cirrhotic PVT. (A1)
2. Acute (recent) PVT is characterized by thrombotic occlusion of the portal vein in the absence of collaterals and cavernous transformation proven by DUS/contrast-enhanced ultrasound (CEUS), CT or MRI. (A1)
3. Malignant PVT is best diagnosed by triphasic CT and DUS/CEUS and characterized by neovascularization of the thrombus, arterial enhancement with rapid washout and direct invasion by an adjacent hepatic mass. (B1)

### Acute non-cirrhotic, non-malignant PVT

4. Patients with acute PVT should receive anticoagulation for at least 6 months to prevent extension to mesenteric veins and intestinal ischemia and in order to achieve recanalization [178–180]. (A1)
5. For acute PVT, LMWH should be initiated and shifted to oral anticoagulation after stabilization of the patient. (A1)
6. In symptomatic patients with acute, non-cirrhotic PVT (i. e. ascites and/or risk of intestinal infarction) a TIPS combined with local clot fragmentation/aspiration should be considered [181]. (B1)
7. Lifelong anticoagulation should be given to PVT patients with a permanent prothrombotic condition. (A1)
8. Long-term anticoagulation is also recommended in patients without identifcation of (prothrombotic) risk factor or thrombus extension into the mesenteric/splenic vein. (B1)
9. For patients with high bleeding risk, therapeutic drug monitoring of LMWH (anti-Xa 0.5–0.8 IU/ml) and of vitamin K antagonists (VKA; International normalized ratio [INR] 2–3) is recommended. (C1)
10. Patients with PVT should be screened for EV and gastric varices. (C1)
11. Large EV and gastric varices should be managed endoscopically before long-term anticoagulation is initiated. (C1)
12. While data on the efficacy and safety of direct oral anticoagulants (DOACs) in patients with PVT are limited, they may be used in patients with non-cirrhotic, non-malignant PVT [3, 178]. (C1)

### Chronic non-cirrhotic, non-malignant PVT

13. For asymptomatic chronic, non-malignant PVT anticoagulation is not indicated. (C1)
14. For symptomatic or progressive chronic, non-malignant PVT anticoagulation should be used. (C1)
15. For chronic, non-malignant PVT associated with a permanent prothrombotic condition anticoagulation should be used. (A1)
16. TIPS may be considered in certain patients with chronic PVT and non-cirrhotic portal hypertension. (B1)
17. Patients with chronic, non-malignant PVT should be screened for EV and gastric varices. (B1)
18. Patients with chronic, non-malignant PVT should receive bleeding prophylaxis before anticoagulation is started. (C1)

### Malignant PVT (regardless of cirrhotic/non-cirrhotic PVT)

19. In general, anticoagulation is not indicated for malignant PVT [178]. (C2)
20. Anticoagulation may be considered for symptomatic and progressive malignant PVT. (C1)
21. TIPS should not be used for treatment of malignant PVT. (C1)

### Acute cirrhotic, non-malignant PVT

22. Anticoagulation is indicated in cirrhotic patients with acute PVT with progression to mesenteric/splenic vein or signs of intestinal ischemia [178]. (A1)
23. Anticoagulation should be considered in all candidates for liver transplantation with PVT [178, 182]. (B1)
24. Anticoagulation may also be used in non-candidates for liver transplantation with progressive PVT or with persisting prothrombotic conditions [176, 178, 182–187]. (C1)
25. No recommendations regarding type of anticoagulation treatment can be made for cirrhotic PVT; however, LMWH and VKA appear to be equally effective for cirrhotic, non-malignant PVT [176, 184–187]. (B1)
26. LMWH should be used as a fixed or weight-adjusted dose. Anti-Xa monitoring of LMWH is not representative in patients with cirrhosis [188]. (C2)
27. VKA should be monitored in patients with cirrhosis with an INR aimed at 2–3 [178]. (C1)
28. Before starting anticoagulation in patients with cirrhotic PVT bleeding prophylaxis should be implemented [178]. (C1)
29. Patients with low platelet count (<50 G/L) are at higher risk of bleeding complications under anticoagulation [3, 178, 187]. (B2)
30. Recent data suggest that DOACs can be safely used in patients with compensated cirrhosis [178, 189]. (C1)
31. TIPS may be considered in selected cirrhotic patients with acute non-malignant ascites. (C1)

## Organizing committee

- Thomas Reiberger
- Peter Fickert (ÖGGH Working Group Liver)
- Andreas Püspök (ÖGGH Working Group Endoscopy)
- Rainer Schöfl (ÖGGH President)
- Maria Schoder (ÖGIR President)

## Writing committee (alphabetical order)

- Theresa Bucsics (Wien): TIPS
- Christian Datz (Oberndorf): Secondary Prophylaxis
- Werner Dolak (Wien): Acute Variceal Bleeding
- Franziska Baumann-Durchschein (Graz): Portal Vein Thrombosis
- Arnulf Ferlitsch (Wien): HVPG Measurement, Acute Variceal Bleeding
- Armin Finkenstedt (Innsbruck): Primary Prophylaxis
- Ivo Graziadei (Hall): Primary Prophylaxis
- Stephanie Hametner (Linz): Diagnosis and Screening of Portal Hypertension
- Franz Karnel (Wien): TIPS, Portal Vein Thrombosis
- Elisabeth Krones (Graz): Hepatorenal Syndrome (AKI-HRS)
- Andreas Maieron (Linz): Diagnosis and Screening of Portal Hypertension, Secondary Prophylaxis
- Mattias Mandorfer (Wien): Pre-primary prophylaxis, Spontaneous Bacterial Peritonitis (SBP), HVPG Measurement, Hepatorenal Syndrome (AKI-HRS)
- Markus Peck-Radosavljevic (Klagenfurt): Spontaneous Bacterial Peritonitis (SBP)
- Florian Rainer (Graz): Management of Ascites
- Maria Schoder (Wien): TIPS, Portal Vein Thrombosis
- Philipp Schwabl (Wien): Portal Hypertensive Gastropathy (PHG), Gastric Varices, HVPG Measurement
- Vanessa Stadlbauer (Graz): Management of Ascites
- Rudolf Stauber (Graz): Management of Ascites
- Heinz Zoller (Innsbruck): Pre-primary prophylaxis, Portal Vein Thrombosis

## Expert panel and review committee (alphabetical order)

- Reto Bale (ÖGIR, Innsbruck)
- Gabriela Berlakovich (Surgery Expert Panel, Wien)
- Herbert Tilg (ÖGGH, Innsbruck)
- Michael Trauner (ÖGGH, Wien)
